# Defective autophagy leads to the suppression of stem-like features of CD271^+^ osteosarcoma cells

**DOI:** 10.1186/s12929-016-0297-5

**Published:** 2016-11-18

**Authors:** Dong Zhang, Qing Zhao, Hao Sun, Lijuan Yin, Jiajun Wu, Jun Xu, Tianxiang He, Chunlei Yang, Chengwei Liang

**Affiliations:** 1 Department of Spinal Disease, Yueyang Hospital of Integrated Traditional Chinese and Western Medicine, Shanghai University of Traditional Chinese Medicine, Shanghai, 200437 People’s Republic of China; 2 Department of Pathology, Changhai Hospital, Second Military Medical University, Shanghai, People’s Republic of China; 3 Department of Orthopedics, Huadong Hospital Affiliated to Fudan University, No. 221 West Yan An Road, Shanghai, 200040 People’s Republic of China

**Keywords:** Osteosarcoma, Autophagy, Cancer stem cells, CD271

## Abstract

**Background:**

As an important stress-response mechanism, autophagy plays crucial role in the tumor formation and drug resistance of cancer cells including osteosarcoma (OS). OS cancer stem cells (CSCs) also are considered a key factor of tumorigenesis, drug resistance and tumor recurrence. However, the relationship between autophagy and OS CSCs still remains unclear.

**Methods:**

CD271+ OS CSCs and CD271- OS cells were isolated by magnetic activated cell sorting. The autophagy level was evaluated by the mRNA expression of autophagy genes, the protein level of LC3II and p62, and the mean number of GFP-LC3 dot per cell. Lentivirus-delivered specific shRNA was utilized to inhibit the corresponding gene expression. The cell viability was examined with CCK8 assay. The cell proliferation level was detected with BrdU staining assay. Cell death was determined by Annexin V/PI double staining of fluorescence activated cell sorting, lactate dehydrogenase release and caspase-3 activity. Tumorigenicity ability was evaluated by colony and sphere formation assay, the protein expression of stemness markers and tumor formation in nude mice.

**Results:**

Our data indicated that CD271+ OS CSCs had a similar basic autophagy level with CD271- OS cells. Autophagy deficiency had no observable effects on the levels of cell proliferation and death both in CD271+ and CD271- OS cells under normal condition. However, CD271+ OS cells showed a higher autophagy activity than CD271- OS cells under hypoxia and low nutrient (LH) condition. Moreover, autophagy-deficient CD271+ OS cells lost the advantage of tolerance to LH condition compared to CD271- OS cells. Meanwhile, autophagy deficiency enhanced the sensitivity to chemotherapeutics in the CD271+ cells to the comparable level in the CD271- cells. More importantly, deficient-autophagy decreased the protein expression of stemness markers and caused the disappearance of the superiority in tumorigenicity in vitro and vivo in CD271+ OS cells.

**Conclusion:**

The results above demonstrated that autophagy contributes to the stem-like features of CD271+ OS CSCs. Inhibition of autophagy is a promising strategy in the CSCs-targeting OS therapy.

**Electronic supplementary material:**

The online version of this article (doi:10.1186/s12929-016-0297-5) contains supplementary material, which is available to authorized users.

## Background

Osteosarcoma (OS) is one of the most common primary malignant bone tumors and mainly affects children, adolescents, and young adults (between the ages of 10 to 25) [1]. OS often occurs in long bones, including the distal femur, proximal tibia and humerus [2]. he current therapy strategy for OS consists of the addition of chemotherapy after surgical removal of tumor, and neoadjuvant chemotherapy followed by surgery. Although combinational chemotherapy has been improved, the five-year survival rate for OS patients still is about 70% [3]. Therefore, novel therapeutic strategy for enhancing the chemotherapy sensitivity of the OS has yet to be explored.

Cancer stem cells (CSCs) are seen as a subpopulation of self-renewing tumor cells, which can differentiate into the daughter tumor cells, have the lower sensitivity to chemotherapy and radiotherapy, and exhibit tumor re-initiating property. Thus, CSCs are deemed to a promising target for cancer therapy. Diverse studies report that OS also has CSCs [4]. For instance, CD133 + [5–7], CD117 + Stro-1 + [8] and Sca-1 + [9] populations in OS cells revealed the CSCs-like characteristics. CD271, an MSC antigen, was likewise identified as an effective OS-CSCs marker. CD271+ cells showed many stem-like features including self-renew, the advantage of forming sarcospheres, drug resistance and tumorigenicity [10].

Macroautophagy (hereafter termed autophagy) is a conserved self-digestive process that serves as a lysosome-dependent degradation and recycling mechanism for providing the biological materials of biosynthesis and energy synthesis. Under stresses, autophagy plays an important role in eliminating redundant or damaged macromolecules, such as proteins, lipids and organelles. Many studies suggest that autophagy contributes to the resistance of tumor cells to sterile microenvironment and chemotherapy [11].

Numerous reports demonstrate that autophagy supports the stemness of CSCs in some types of tumors, including breast cancer [12–14], pancreatic ductal adenocarcinoma [15, 16], colon cancer [17, 18], hepatocarcinoma [19] and bladder cancer [20]. On the other hand, various researches also reveal that under some conditions, CSCs have a lower autophagy level than non-CSCs [21–23]. Meanwhile, Yujie Fu and his colleagues indicated that autophagy contributed to the resveratrol-induced decrease of CSCs in breast cancer [24]. Liu S, et al. also reported that inhibition of autophagy rescued the reduction of CSCs induced by Ginsenoside rh2 treatment [25]. These researches showed the complexity of the roles of autophagy in CSCs. Thus, in this study, we investigated the influence of autophagy on OS CSCs by detecting whether and how autophagy inhibition impacts on CD271+ OS CSCs.

## Methods

### Cell culture

The SAOS2 and MNNG/HOS human OS cell lines were obtained from the American Type Culture Collection and Cell Bank of Chinese Academy of Sciences, respectively. SAOS2 cells were cultured in McCoy’s 5A medium (GIBCO, Invitrogen, Carlsbad, CA) supplemented with 10% fetal bovine serum (FBS, GIBCO). MNNG/HOS cells were maintained in Dulbecco’s modified Eagle’s medium (DMEM, GIBCO) supplemented with 10% FBS. All cells were grown in 37 °C under humidified air containing 5% CO_2_.

Low nutrients medium (glucose 100 mg/L, no L-glutamine) was mixed by low glucose DMEM (GIBCO, No. 11880036) and no glucose DMEM (GIBCO, No. A14430-01) at the ratio of 1:9. For hypoxia condition, cells were cultured in a tri-gas incubator (SANYO, Osaka, Japan) maintained at 1% O_2_, 94% N_2_ and 5% CO_2._ The low nutrients and hypoxia (LH)-treated cells were cultured in normal condition for overnight, and were cultured under LH condition for the indicated times.

### Magnetic activated cell sorting (MACS)

CD271+ and CD271- OS cells were sorted using CD271 MicoBead Kit (Order no. 130-092-819, Miltenyi Biotec. Auburn, CA) depending on its manual. Briefly, OS cells were collected and dissociated into single-cell suspension with MACS separation Buffer. OS cells were incubated with FcR Blocking Reagent and CD271-PE antibody in 4 °C for 10 min. Then, FcR Blocking Reagent and Anti-PE MicroBeads were added and mixed at 4 °C for 15 min. After washed with PBS, CD271+ OS cells were sorted with magnetic separation columns and MACS separator. The quality of MACS was checked with flow cytometry (Order no.130-110-115, Miltenyi Biotec.) on a BD Fluorescence Activated Cell Sorting (FACS) Calibur machine (BD Biosciences, San Jose, CA).

### FACS

The indicated OS cells were dissociated into single-cell suspension. 10^5^ cells/sample was added with VioBright FITC-labeled CD271 antibody (Order no.130-110-115, Miltenyi Biotec.) or corresponding isotype controls and were incubated in 4 °C for 30 min. After washed twice with PBS, these cells were analyzed with a BD FACS Calibur machine (BD Biosciences).

### Quantitative real-time polymerase chain reaction (qPCR)

Total RNA was extracted from the indicated OS cells using an Ultra-Pure Total RNA QuickExtract Kit (BioTeke corporation, Beijing, China) according to its protocol. RNA concentrations were quantified with a ND-2000 spectrophotometer (Nanodrop Technologies, Thermo Scientific, Wilmington, DE). Then, RNA was reverse transcribed with a RT Reagent Kit (TaKaRa, Tokyo, Japan) to generate complementary DNA. The qPCR was performed in a Roche LightCycler® 480 System utilizing regular 2-step qPCR program. The specific primers were purchased from QIAGEN: Beclin1, Product no. PPH05670B; LC3B, Product no. PPH17765B; Atg5, Product no. PPH07779A; Atg7, Product no. PPH15687C; GAPDH, Product no. PPH00150F. The relative mRNA expressions of the indicated genes were calculated using the ΔΔCt method, and GAPDH was used for normalization.

### Western blot

The indicated OS cells were harvested and lysed in protein extraction reagent (Pierce, Thermo Scientific) with cocktail (Roche Life Science, Indianapolis, IN). Samples containing equal amounts of protein were subjected to SDS-PAGE. The blots were incubated with the primary antibodies against LC3, SQSTM1/p62, Atg5, Atg7, Nanog, Oct4, Stat3, p-Stat3 (all from Cell Signaling Technology, Beverly, MA), GAPDH and β-actin (both from Hangzhou HuaAn Biotech, Hangzhou, Zhejiang, China), and then with the secondary antibodies labeled peroxidase (Hangzhou HuaAn Biotech) and chemiluminescent substrates. GAPDH and β-actin served as control proteins.

### GFP-LC3 transfection

The GFP-LC3-carrying adenovirus (Hanbio, Shanghai, China) was used to transfected the indicated OS cells according to the manufacturer’s protocol. After the indicated treatments, autophagy level was examined with counting the number of GFP-LC3 punta per cells under a fluorescence microscope (Olympus IX73, Melville, NY). A minimum of 100 cells per sample was calculated in three replicates for each experiment.

### Short hairpin RNA (shRNA)-mediated gene silencing

The indicated OS cells were transfected with lentivirus vector expressing Atg5-shRNA (5’-G ATTCATGGAATTGAGCCAAT-3’), Atg7-shRNA (5’-CCAGAGAGTTTACCTCTCATT-3’) or scramble (Scr)-shRNA (5’-TTCTCCGAACGTGTCACGT-3’) (all from Shanghai GeneChem, Shanghai, China). After 2 days, the transfected cells were selected by puromycin (2 mg/ml, Sigma, St. Louis, Mo) for 48 h.

### Cell Counting kit-8 (CCK8) assay

The cell viability was detected with CCK8 assay (Beyotime Institute of Biotechnology, Suzhou, Jiangsu, China). After the indicated treatments, CCK8 solution (10 μl) was added in each well (96-well plates). Then, the indicated OS cells were cultured at 37 °C for another 1 h. The optical density of each well was measured at 450 nm with a Bio-Tek microplate reader (Bio-Tek Instruments, Thermo Fisher Scientific, Winooski, VT).

### 5-Bromo-2-deoxyUridine (BrdU) staining

BrdU (10 μM, Sigma) was added to the culture media of the indicated OS cells. After 24 h, the cells were fixed in 4% paraformaldehyde for 10 mins, and permeabilized in 0.1% Triton X-100 for 5 mins at room temperature. DNA of these cells was denatured by HCl (2 M) for 20 mins at 37 °C. Then, these cells were neutralized by borate buffer (0.1 M, pH 8.5) for 5 mins three times. BrdU was detected by anti-BrdU IgG1 conjugated to Alexa Fluor 555 (Invitrogen), and nuclei were stained with DAPI.

### Detection of cell death by Annexin V/PI double staining

After the indicated treatments, cells were stained by FITC-labeled Annexin V and PI (Nanjing Keygen Biotech, Nanjing, Jiangsu, China) depending to its protocol. FACS analysis for Annexin V/PI double staining was done with a BD FACS Calibur machine (BD Biosciences). Dead cells consisted of PI+ cells (Q1), PI + FITC+ cells (Q2) and FITC+ cells (Q4). All experiments were carried out three times.

### Colony formation assay

The indicated cells were seeded in McCoy’s 5A or DMEM with 1% FBS at a density of 400 cells/well on 6-well plates. After 10 days, these plates were stained with crystal violet (Beyotime Institute of Biotechnology) and counted the number of colonies per well. All experiments were performed three times.

### Tumor sphere formation assay

The indicated cells were seeded in McCoy’s 5A or DMEM with 10 ng/ml bFGF, 20 ng/ml EGF and 2% B27 (Invitrogen) at a density of 1000 cells/well on ultra low attachment 6-well plates. Fresh equal amount of bFGF and EGF were added every 2 days. After 10 days, the number of tumor spheres containing at least 50 cells per well was observed and counted by a microscopy.

### Tumor formation in vivo

Male BALB/c nude mice (6 weeks old, weighing 18–20 g) were purchased from Shanghai Experimental Center, Chinese Science Academy, Shanghai. The mice were kept at a specific pathogen-free animal facility. All procedures of animal treatments followed the guidelines issued by National Institute of Health and this study was approved by the Animal Ethics Committee of Shanghai University of Traditional Chinese Medicine (TCM-2015-018-E20).

The indicated cells of different numbers were suspended in McCoy’s 5A or DMEM with an equal volume (100 μl) of Matrigel (BD Biosciences) and injected subcutaneously into the oxter of left forelimb of BALB/c nude mice. In the Chloroquine (CQ, sigma) combination groups, the mice were received intraperitoneal injection of autophagy inhibitor CQ (60 mg/kg) once every three days. The volume of subcutaneous tumors in mice was monitored once a week for 6 weeks. The tumors were measured with electronic calipers and tumor volume was estimated using the formula 1/2*a*b^2^, where ‘a’ and ‘b’ were the longest and shortest diameters of the tumor, respectively. After 6 weeks, the mice that had a tumor (diameters ≥ 3 mm) were considered the tumor-bearing mice. And the number of tumor-bearing mice of every group was counted. The tumors were soaked in 10% neutral-buffered formalin for paraffin embedding and subsequent hematoxylin and eosin (H&E) staining.

### Detection of lactate dehydrogenase (LDH) release

The LDH release was quantified with a LDH Cytotoxicity Detection Kit (Roche Applied Science, Mannheim, Germany) according to the manufacturer’s protocol. After the indicated treatments, 100 μl of reaction cocktail was added to 100 μL of culture medium and was incubated for 30 mins at room temperature. Then, the LDH release was tested by measuring the absorbance at 490 nm with a Bio-Tek microplate reader (Bio-Tek Instruments). The value of LDH release was established with the following formula: LDH release = (exp. value - low control)/(high control - low control). All treatment conditions were performed in six replicate and all experiments were repeated three times.

### Detection of caspase-3 activity

The caspase-3 activity was quantified with an APOPCYTO Caspase-3 Colorimetric Assay Kit (MBL International Corp., Nagoya, Japan) according to the manufacturer’s instruction. In brief, the indicated cells were harvested and lysed. Cell lysate (20 μl) was added to the buffer containing Ac-DEVD-pNA to yield a 100 μl total reaction volume, and the mixture was incubated for 1 h at 37 °C. Optical density of each well was evaluated at 405 nm using a Bio-Tek microplate reader (Bio-Tek Instruments). The concentrations of released pNA were quantified based on the absorbance and calibration curve. The caspase-3 activity in the control group is deemed as 1.

### Drug sensitivity assessment

The indicated cells were seeded in 96-well plates at a density of 5000 cells/well. After overnight, cells were treated with Cisplatin or Epirubicin at different concentrations (Cisplatin, 0–8 μg/ml; Epirubicin, 0–5 μg/ml) for 48 h. The cell viability was assessed with CCK8 assay to determine the IC50 value.

### Statistics

Data are shown as the means ± S.D. Statistical significance was determined with One-way ANOVA using GraphPad Prism 6.04 software. For all tests, A value of *P* < 0.05 was considered statistically significant.

## Results

### CD271+ OS cells have higher autophagy ability rather than basic autophagy level compared to CD271- OS cells

**Fig. 1:**
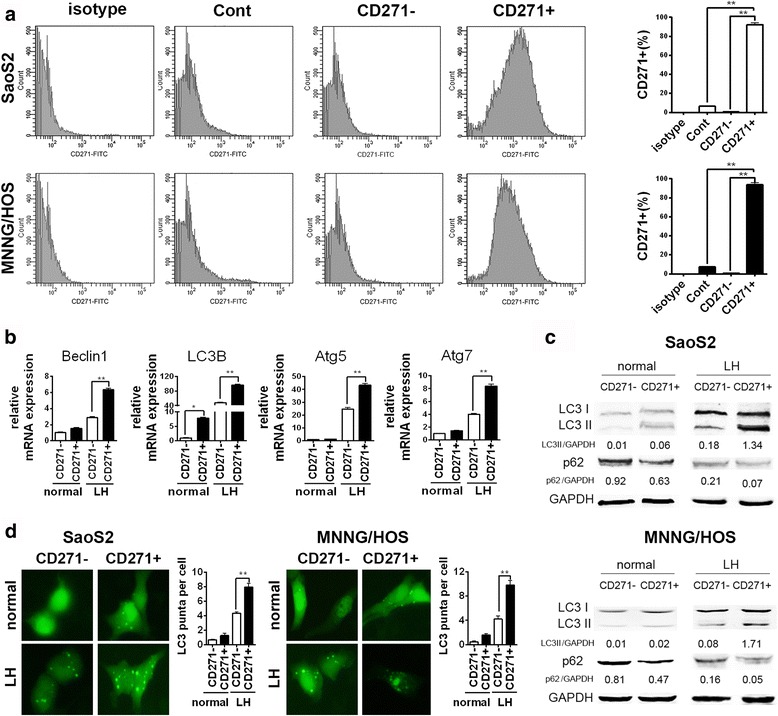
CD271+ OS cells have higher autophagy level than CD271- OS cells under LH condition. **a** CD271+ and CD271- OS cells were separated by MACS. Then, the percentage of CD271+ cells in the indicated OS cells was detected with FACS assay. The data are shown as the mean ± S.D. (*n* = 3; **, *P* < 0.01). **b** The mRNA expression of autophagy-relative genes in the CD271- and CD271+ SaoS2 cells under normal and LH conditions at 24 h was examined with qPCR. The data are shown as the mean ± S.D. (*n* = 3; **, *P* < 0.01). **c** The protein level of LC3 and p62 in the indicated OS cells under normal and LH conditions at 24 h was detected by western blotting. **d** The GFP-LC3-transfected OS cells under normal and LH conditions at 24 h were captured with a fluorescence microscope. The mean number of GFP-LC3 dots per the indicated OS cells from three replicates are shown as the mean ± S.D. (**, *P* < 0.01)

To study the role of autophagy in OS CSCs, we isolated CD271+ OS cells and CD271- OS cells with MACS and examined their purities with FACS (Fig. 1a). Then, we explored whether there is the difference in autophagy level between CD271+ and CD271- OS cells. Firstly, we detected the basic mRNA expression of various autophagy-essential genes, including Beclin1, LC3B, Atg5 and Atg7. However, only the basic mRNA expression of LC3B showed a higher level in CD271+ OS cells opposed to that in CD271- OS cells (Fig. 1b). We further examined the mRNA expression of these genes in CD271+ and CD271- OS cells after autophagy inducer treatment. Both nutrient deficiency and hypoxia are the classical features of tumor microenvironment, and can induce autophagy [11]. The results revealed that the mRNA expression of all four genes in CD271+ OS cells had a higher level than those of CD271- OS cells after LH treatment (Fig. 1b). Western blot assay showed similar results. After LH treatment, CD271+ OS cells showed higher LC3II level and lower p62 level compared to CD271- OS cells (Fig. 1c). Moreover, LC3 dots, an indicator of autophagosome formation, also had a higher number in CD271+ OS cells than that in CD271- OS cells under LH condition (Fig. 1d). These data demonstrated that although there is no significant difference in the basic autophagy level between CD271+ OS CSCs and CD271- OS cells, CD271+ OS CSCs have higher autophagy activity than CD271- OS cells under LH condition.

### The basic biological activities in both CD271+ and CD271- OS cells are not significantly affected by autophagy deficiency under normal condition

**Fig. 2:**
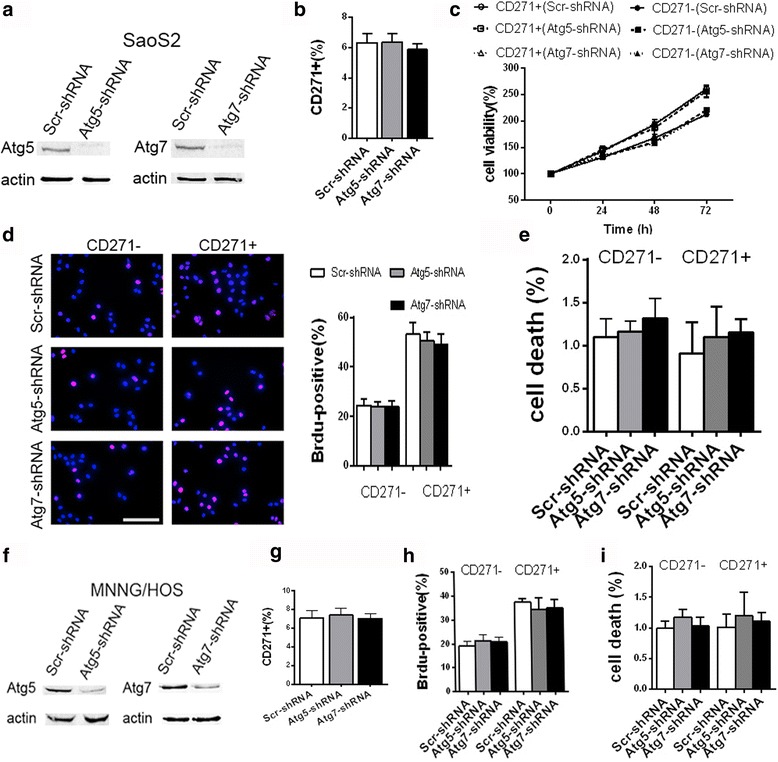
Autophagy-deficiency has no significant influence on basic biological activities in OS cells under normal condition. **a**–**i** The indicated OS cells were cultured in normal condition. **a**, **f** The protein level of Atg5 and Atg7 in the transfected SaoS2 (**a**) and MNNG/HOS (**f**) cells was detected by western blotting. **b**, **g** The percentage of CD271+ cells in the transfected SaoS2 (**b**) and MNNG/HOS (**g**) cells were examined with FACS assay. The data are shown as the mean ± S.D. (*n* = 3). **c** The cell viability of the indicated SaoS2 cells was detected with CCK8 assay at the indicated time points. The data are shown as the mean ± S.D. (*n* = 3). **d**, **h** The indicated SaoS2 cells were stained with BrdU and images were captured with a fluorescence microscope. The percentage of Brdu + cells in the indicated SaoS2 (**d**) and MNNG/HOS (H) cells was shown in graphs. The data from three replicates are shown as the mean ± S.D. **e**, **i** The cell death of the indicated SaoS2 (**e**) and MNNG/HOS (**i**) cells was analyzed by Annexin-V-FITC/PI double staining with FACS. The percentage of dead cells (Annexin-V-positive or PI-positive) was shown in graph. The data from three replicates are shown as the mean ± S.D

OS CSCs had various stem-like features, such as the resistances to ischemic microenvironment and chemotherapeutics, and high tumorigenicity in vitro and vivo [4]. We need to confirm the influences of autophagy deficiency on the basic biological activities in OS CSCs and OS non-CSCs before evaluating the impacts of autophagy deficiency on these stemness features of OS CSCs. Thus, we used specific shRNA to disturb expression of Atg5 or Atg7 in SaoS2 OS cells (Fig. 2a), and investigate the impact of autophagy inhibition on the basic biological activity of SaoS2 cells under normal condition. FACS analysis exhibited that inhibition of autophagy did not result in the remarkable change of the percentage of CD271+ cells in SaoS2 cells (Fig. 2b). CCK8 assay also showed that autophagy deficiency had no significant influence on cell viability both in CD271+ and CD271- SaoS2 cells (Fig. 2c). The results of BrdU staining and Annexin V/PI double staining further suggested that autophagy deficiency had no obvious influences on cell proliferation and death both in CD271+ and CD271- SaoS2 cells (Fig. 2d and e). Moreover, these detections in MNNG/HOS OS cells also obtained similar results (Fig. 2f–i). These data demonstrated that autophagy deficiency had no prominent impacts on the basic biological activities both in CD271+ and CD271- OS cells.

### Autophagy-deficient CD271+ OS cells lose the advantage of tolerance to LH condition

**Fig. 3:**
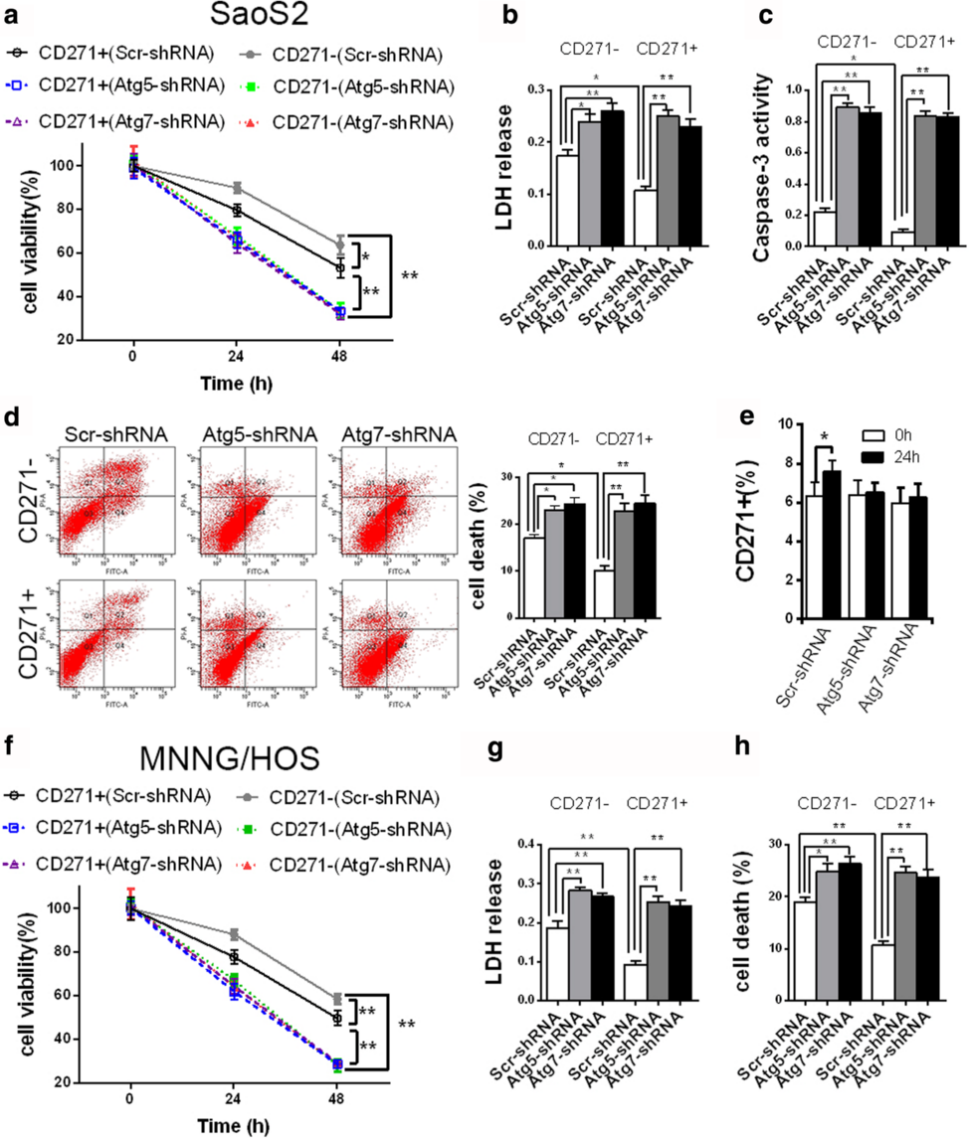
Autophagy-deficiency reduces the advantages of CD271+ OS cells in terms of resistance to LH condition. **a**–**h** The indicated OS cells were cultured in LH condition. **a**, **f** The cell viability of the indicated SaoS2 (**a**) and MNNG/HOS (**f**) cells was detected by CCK8 assay at the indicated time points. The data are shown as the mean ± S.D. (*n* = 3; *, *P* < 0.05; **, *P* < 0.01). **b**, **g** The LDH release level of the indicated SaoS2 (**b**) and MNNG/HOS (**g**) cells under LH treatment was detected at 24 h. The data are shown as the mean ± S.D. (*n* = 3; *, *P* < 0.05; **, *P* < 0.01). **c** The caspase-3 activity of the indicated SaoS2 cells under LH treatment was detected at 24 h. The data are shown as the mean ± S.D. (*n* = 3; *, *P* < 0.05; **, *P* < 0.01). **d**, **h** The cell death of the indicated SaoS2 (**d**) and MNNG/HOS (H) cells under LH treatment was analyzed by Annexin-V-FITC/PI double staining with FACS. The percentage of dead cells (Annexin-V-positive or PI-positive) was shown in graph. The data from three replicates are shown as the mean ± S.D. **e** The percentage of CD271+ cells in the transfected SaoS2 cells under LH treatment at the indicated times was examined with FACS assay. The data are shown as the mean ± S.D. (*n* = 3)

Numerous studies have reported that CSCs have more resistance to barren microenvironment [4]. Our data of CCK8 assay suggested that CD271+ SaoS2 cells had higher cell viability than CD271- SaoS2 cells under LH culture condition, but inhibition of autophagy reversed this trend (Fig. 3a). Detection of LDH release further confirmed these data. The level of LDH release in CD271+ SaoS2 cells was more than that in CD271- SaoS2 cells under LH condition. And autophagy defects lead to increased LDH release both in CD271+ and CD271- SaoS2 cells. Moreover, there was not any noticeable difference in LDH release level between autophagy-deficient CD271+ and CD271- SaoS2 cells (Fig. 3b). Detections of another two cell death evaluation assays, caspase-3 activity and flow cytometry analysis using Annexin V/PI double staining, obtained similar results (Fig. 3c and d). FACS analysis also suggested that LH treatment resulted in the increase of percentage of CD271+ cells in SaoS2 cells, but autophagy deficiency reversed this increase (Fig. 3e). We further verified these results in MNNG/HOS cells (Fig. 3f–h). These data demonstrated that autophagy-deficient CD271+ OS cells lost the advantage of tolerance to LH condition.

### Autophagy deficiency results in the elimination of the advantage of drug resistance in CD271+ OS cells

**Fig. 4:**
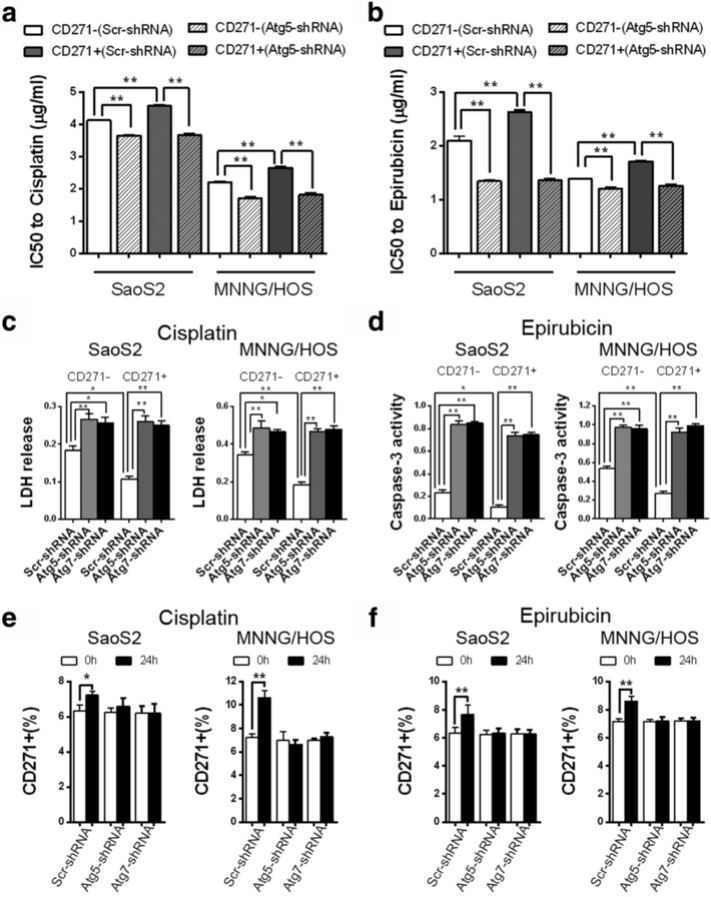
Autophagy-deficiency eliminates the advantages of CD271+ OS cells in terms of resistance to chemotherapeutics. **a**, **b** The indicated SaoS2 and MNNG/HOS cells were treated with Cisplatin or Epicubicin of different doses for 48 h, and IC50 of the indicated OS cells to Cisplatin (**a**) or Epicubicin (**b**) were evaluated by CCK8. The data are shown as the mean ± S.D. (*n* = 3; **, *P* < 0.01). **c** The LDH release level of the indicated SaoS2 and MNNG/HOS cells was detected at 24 h after Cisplatin (4 μg/ml) treatment. The data are shown as the mean ± S.D. (*n* = 3; *, *P* < 0.05; **, *P* < 0.01). **d** The caspase-3 activity of the indicated SaoS2 and MNNG/HOS cells was detected at 24 h after Epirubicin (2 μg/ml) treatment. The data are shown as the mean ± S.D. (*n* = 3; *, *P* < 0.05; **, *P* < 0.01). **e**, **f** FACS assay detected the percentage of CD271+ cells in the indicated SaoS2 and MNNG/HOS cells at 24 h after Cisplatin (4 μg/ml, **e**) or Epirubicin (2 μg/ml, **f**) treatment. The data are shown as the mean ± S.D. (*n* = 3; *, *P* < 0.05; **, *P* < 0.01)

Drug resistance of CSCs is considered an important cause resulting in chemotherapy- resistant and recurrence of tumor. We used Cisplatin of different concentration to treat CD271+ and CD271- OS cells for 48 h. The results revealed that CD271+ OS cells had a higher IC50, an indicator of drug resistance, to Cisplatin compared with CD271- OS cells. Autophagy deficiency increased substantially IC50 to Cisplatin both in CD271+ and CD271- OS cells. Importantly, autophagy-deficient CD271+ OS cells had an approximate IC50 to Cisplatin with autophagy-deficient CD271- OS cells (Fig. 4a and Additional file 1A). In the Epirubicin treatment, we observed similar results (Fig. 4b and Additional file 1B). Detections of LDH release and caspase-3 activity also showed that autophagy deficiency resulted in the disappearance of superiority of drug resistance in CD271+ OS cells (Fig. 4c and d). After Cisplatin or Epirubicin treatment, higher drug resistance of CD271+ OS cells led to increased percentage of CD271+ cells, but inhibition of autophagy reversed this trend (Fig. 4e and f). The results above demonstrated that autophagy deficiency brought about the elimination of advantage of drug resistance in CD271+ OS cells.

### Defective autophagy leads to the loss of superiority of tumorigenicity in CD271+ OS cells

**Fig. 5:**
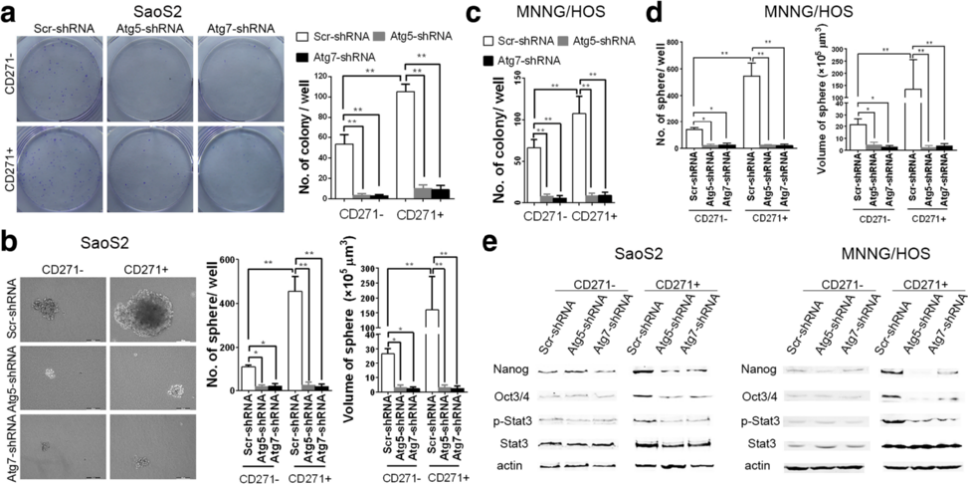
Autophagy-deficiency decreases the advantage of CD271+ OS cells in terms of tumorigenesis in vitro. **a**–**d** The colony-forming and sphere-forming abilities of the indicated SaoS2 (**a**, **b**) and MNNG/HOS (**c**, **d**) cells were detected. The mean number of colonies (**a**, **c**) and spheres (**b**, **d**) per well and the mean volume of spheres in the indicated groups are shown in graphs. The data from three replicates are shown as the mean ± S.D. **e** The protein expression of Nanog, Oct3/4, Stat3 and p-Stat3 in the indicated OS cells were examined by western blotting

CSCs have the advantage of tumorigenicity in vitro and vivo compared to non-CSCs. We next detected the influence of autophagy deficiency on tumorigenicity of CD271+ OS cells. Colony formation assay showed that CD271+ SaoS2 cells had more colony formation than CD271- SaoS2 cells. And autophagy deficiency reduced colony formation ability both in CD271+ and CD271- SaoS2 cells. Moreover, there was no remarkable difference in colony formation ability between autophagy-deficient CD271+ and CD271- SaoS2 cells (Fig. 5a). In Fig. 2, our results showed that autophagy deficiency had no obvious impact on cell proliferation and death of CD271+ OS cells. Therefore, the result of Fig. 5a suggested that autophagy deficiency declined significantly tumorigenicity in vitro of CD271+ SaoS2 cells. To further confirm this finding, we also utilized tumor sphere formation assay. The results showed that CD271+ SaoS2 cells had more sphere formation and larger sphere volume compared to CD271- SaoS2, but autophagy deficiency reversed this trend (Fig. 5b). Meanwhile, we observed similar consequences in MNNG/HOS cells (Fig. 5c and d). Moreover, autophagy deficiency decreased the protein level of stemness factors Nanog, Oct3/4 and p-Stat3 [10, 26] in CD271+ OS cells to the comparable level in CD271- OS cells (Fig. 5e).

**Fig. 6:**
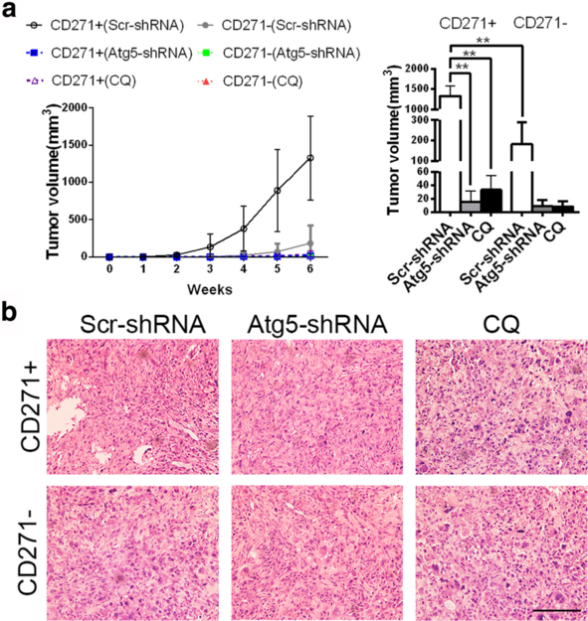
Autophagy inhibition restricts the advantage of CD271+ OS cells in terms of tumorigenesis in vivo. **a** Growth curve of tumor originated from 10^7^ indicated MNNG/HOS cells in 6 weeks (*left panel*). Tumor volumes of the indicated groups at 6 weeks are shown at the right panel. The data are shown as the mean ± S.D. (*n* = 5; **, *P* < 0.01). **b** Representative H&E staining images in tumors originated from 10^7^ indicated MNNG/HOS cells at 6 weeks. Magnification, 200×. Bar, 100 μm

We further examined the influence of autophagy inhibition on tumorigenicity in vivo of CD271+ cells. We observed that autophagy inhibition resulted in the elimination of advantage of tumor growth in CD271+ OS cells (Fig. 6a). The results revealed that just 1 × 10^6^ CD271+ cells were large enough to form a tumor, but tumor incidence of 1 × 10^7^ CD271- cells only was three-fifths. Tumor incidence of 1 × 10^7^ Atg5-deficient CD271+ cells was reduced to one-fifths. CQ treatment was also decreased tumor incidence of CD271+ cells. Autophagy-inhibited CD271+ cells had similar tumor incidence with autophagy-inhibited CD271- cells (Table 1). Additionally, both in the tumors originated from CD271+ and CD271- OS cells, autophagy inhibition had no obvious influence on the tumor tissue morphology (Fig. 6b). All data above demonstrated that autophagy inhibition resulted in the loss of superiority of tumorigenicity in CD271+ OS cells.

**Table 1:** Tumor incidence of CD271+ or CD271- transfected MNNG/HOS cells in vivo

Cell number (per 100 μl)	1 × 10^5^	1 × 10^6^	1 × 10^7^
CD271 + (Scr-shRNA)	2/5	4/5	5/5
CD271 + (Atg5-shRNA)	0/4	0/5	1/5
CD271 + (CQ)	0/4	0/5	2/5
CD271-(Scr-shRNA)	0/4	0/4	3/5
CD271-(Atg5-shRNA)	0/4	0/4	1/5
CD271-(CQ)	0/4	0/4	1/5

MNNG/HOS cells were injected subcutaneously into the oxter of left forelimb of BALB/c-Nude mice. Numbers indicate tumor bearing/injected mice

## Discussion

In this study, we found that CD271+ OS CSCs had a similar basic autophagy level with CD271- OS cells, but CD271+ cells had a higher autophagy activity than CD271- cells under LH condition. Autophagy deficiency had no prominent effects on cell proliferation and death both in CD271+ and CD271- OS cells. However, stemness of CD271+ OS cells was significantly fell by inhibiting autophagy. CD271+ OS cells had numerous advantages, including resistances to LH condition and chemotherapeutics and tumorigenicity, compared to CD271- OS cells. Autophagy deficiency obviously suppressed the three characteristics of CD271+ OS cells. Autophagy-deficient CD271+ OS cells had no remarkable difference with autophagy-deficient CD271- OS cells in these three stemness-associated respects. These findings suggest that autophagy contributes to the stemness of CD271+ OS cells.

Previous studies demonstrated that autophagy is a protective mechanism of OS cells. Huang J, et al. found that HMGB1 increased drug resistance of OS cells by inducing autophagy [27]. Shimizu T and his colleagues also reported that IGF2-induced autophagy decreased chemotherapeutic sensitivity in OS cells [28]. Autophagy induced by PERK, an ubiquitously-expressed endoplasmic reticulum (ER) protein kinase, contributed to the resistance to ER stress-induced apoptosis in OS cells [29]. Both hypoxia and nutrient deficiency induced reactive oxygen species (ROS) production [30]. The research of Liu Y, et al. suggested that DSTD, an androstenedione derivative, induced ROS-mediated autophagy. And autophagy inhibition promoted cell death by DSTD treatment [31]. In addition, the study of Akin D, et al. reveal that suppression of autophagy by an ATG4B antagonist reduced the development of SaoS-2 OS tumors in vivo [32]. These studies are in accord with our findings that autophagy deficiency increased the sensitivity to LH condition and chemotherapy drugs and reduced tumorigenesis both in CD271+ and CD271- OS cells. And our findings indicated that autophagy plays a crucial role in these aspects of OS cells because there is no significant difference in these aspects in autophagy-deficient CD271+ and CD271- cells. Notably, some earlier studies showed that autophagy increases the sensitivity to chemotherapeutics in OS cells by autophgic death. For example, Meschini S, et al. reported that bisindolic alkaloid voacamine induced autophagy was effective against drug resistance tumor cells in vitro [33]. These research hints that the influence of autophagy inhibition on drug resistance of OS cells has complexity and may involve the drug specificity for autophagy and overall influences of autophagic protection and autophagic death.

Compared with non-CSCs, CSCs have higher autophagy level in breast cancer [13], pancreatic ductal adenocarcinoma [15], hepatoma [19] and bladder cancer [20], but lower autophagy level in glioblastoma [21]. In our study, we found that CD271+ OS CSCs had similar basic autophagy level and higher autophagy activity compared to CD271- OS cells. Meanwhile, numerous researches investigated the influence of autophagy on CSCs. Two research teams found that autophagy contributes to maintenance of CD44 + CD24-/low CSCs and ALDH+ CSCs [12–14]. Research on pancreatic ductal adenocarcinoma revealed that moderate autophagy helped CSCs survival under hypoxia starvation condition, but both autophagy inhibition and excess autophagy resulted in killing CSCs [15]. Research of Wei Lixin team demonstrated that autophagy played a major role in the survival of liver CSCs under hypoxia starvation microenvironment [19]. Yang Hao-Zheng, et al. reported that autophagy enhanced chemoresistance of colorectal CSCs [18]. However, Stephen Doxsey and his colleagues reported that autophagy inhibition promoted malignant transformation of stem cells by inducing midbody accumulation [34]. And two studies showed that autophagy contributes to drug-induced decrease of CSCs [24, 25]. The different role of autophagy on CSCs is possibly due to various kinds of cancers and specific impact factors. Additionally, our results revealed that autophagy deficiency decreases stemness of CD271+ OS cells, but have no prominent influence on percentage of CD271+ OS cells. This finding hints CD271+ OS cells may have a cell subpopulation which has more features of CSCs.

## Conclusion

In summary, our results indicated that CD271+ OS CSCs had higher autophagy activity than CD271- OS cells under stress condition. And autophagy contributes to the stemness of CD271+ OS CSCs. Autophagy-deficient CD271+ OS CSCs disappeared the advantages in terms of tolerance to LH condition, drug resistance and tumorigenicity in vitro and vivo compared to CD271- OS cells.

## Additional file

Additional file 1: Cell viaiblities of OS cells after chemotherapeutics treatments. (A, B) The indicated SaoS2 and MNNG/HOS cells were treated with Cisplatin (A) or Epicubicin (B) of different doses for 48 h. Then, the cell viability of the indicated cells was detected by CCK8 assay. The data are showen as the mean ± S.D. (n = 3). (PPTX 232 kb)

## Abbreviations

BrdU: 5-Bromo-2-deoxyUridine
CCK8: Cell counting kit-8
CQ: Chloroquine
CSCs: Cancer stem cells
DMEM: Dulbecco’s modified Eagle’s medium
ER: Endoplasmic reticulum
FACS: Fluorescence activated cell sorting
FBS: Fetal bovine serum
H&E: Hematoxylin and eosin
LDH: Lactate dehydrogenase
LH: Low nutrients and hypoxia
MACS: Magnetic activated cell sorting
OS: Osteosarcoma
qPCR: Quantitative real-time polymerase chain reaction
ROS: Reactive oxygen species.
Scr: Scramble
shRNA: Short hairpin RNA
