# Supplementary figures and images for: Defective autophagy leads to the suppression of stem-like features of CD271+ osteosarcoma cells
Source: J Biomed Sci. 2016 Nov 18;23:82. doi: 10.1186/s12929-016-0297-5 (PMC5116184; doi:10.1186/s12929-016-0297-5)

## Slide 1
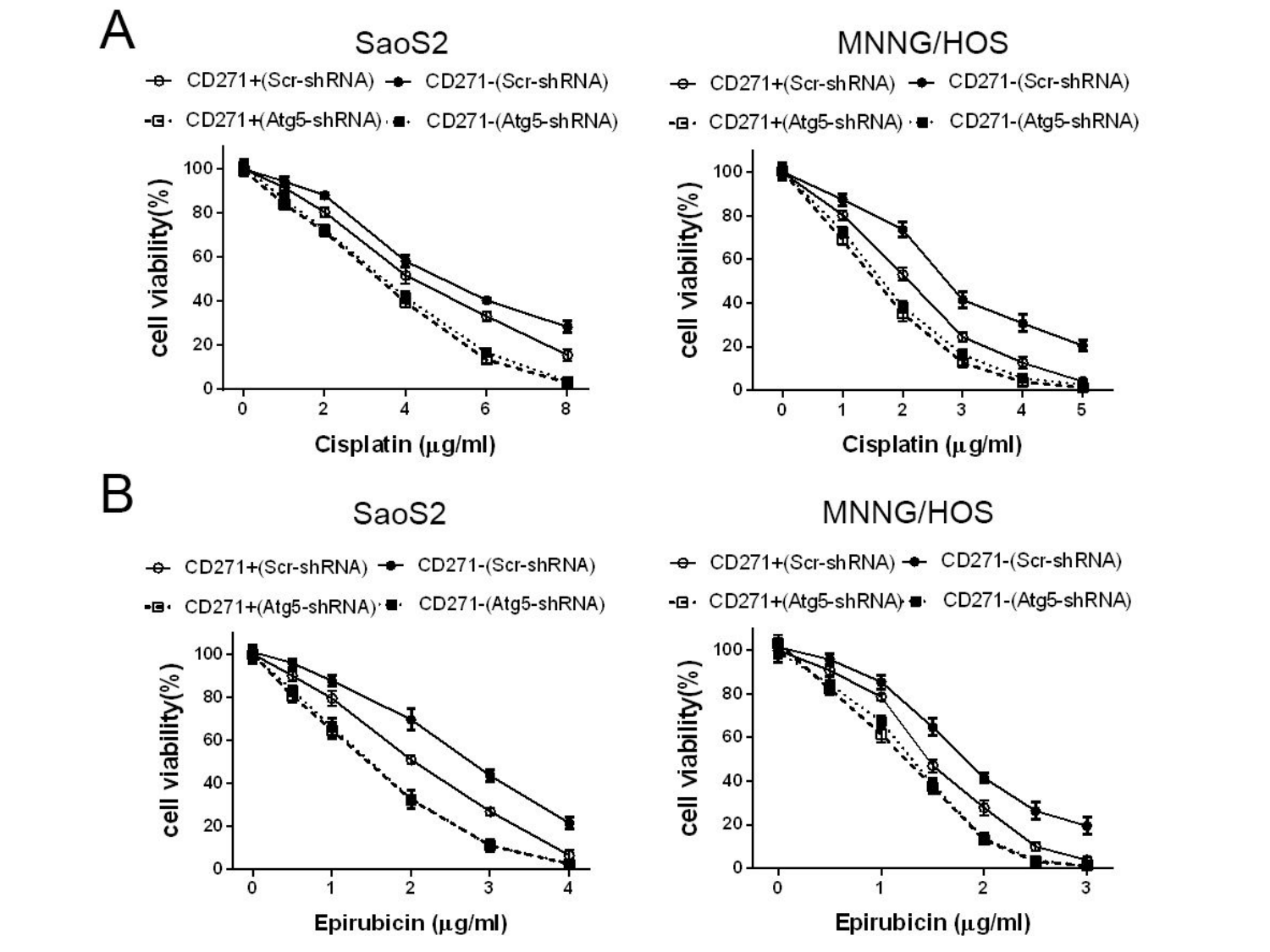

Supplement: Additional file 1: — Cell viaiblities of OS cells after chemotherapeutics treatments. (A, B) The indicated SaoS2 and MNNG/HOS cells were treated with Cisplatin (A) or Epicubicin (B) of different doses for 48 h. Then, the cell viability of the indicated cells was detected by CCK8 assay. The data are showen as the mean ± S.D. (n = 3). (PPTX 232 kb) [file 12929_2016_297_MOESM1_ESM.pptx]
